# One Health: navigating plague in Madagascar amidst COVID-19

**DOI:** 10.1186/s40249-023-01101-3

**Published:** 2023-05-16

**Authors:** Ritik Agrawal, Jogesh Murmu, Sweta Pattnaik, Srikanta Kanungo, Sanghamitra Pati

**Affiliations:** ICMR-Regional Medical Research Centre, Bhubaneswar, Odisha India

**Keywords:** Plague, Madagascar, WASH, Surveillance, Animal surveillance, One Health, *Yersinia pestis*, Early warning

## Abstract

**Background:**

Africa sees the surge of plague cases in recent decades, with hotspots in the Democratic Republic of Congo, Madagascar, and Peru. A rodent-borne scourge, the bacterial infection known as plague is transmitted to humans via the sneaky bites of fleas, caused by *Yersinia pestis*. Bubonic plague has a case fatality rate of 20.8% with treatment, but in places such as Madagascar the mortality rate can increase to 40–70% without treatment.

**Main text:**

Tragedy strikes in the Ambohidratrimo district as three lives are claimed by the plague outbreak and three more fight for survival in the hospitals, including one man in critical condition, from the Ambohimiadana, Antsaharasty, and Ampanotokana communes, bringing the total plague victims in the area to a grim to five. Presently, the biggest concern is the potential plague spread among humans during the ongoing COVID-19 pandemic. Effective disease control can be achieved through training and empowering local leaders and healthcare providers in rural areas, implementing strategies to reduce human–rodent interactions, promoting water, sanitation and hygiene practices (WASH) practices, and carrying out robust vector, reservoir and pest control, diversified animal surveillance along with human surveillance should be done to more extensively to fill the lacunae of knowledge regarding the animal to human transmission. The lack of diagnostic laboratories equipped represents a major hurdle in the early detection of plague in rural areas. To effectively combat plague, these tests must be made more widely available. Additionally, raising awareness among the general population through various means such as campaigns, posters and social media about the signs, symptoms, prevention, and infection control during funerals would greatly decrease the number of cases. Furthermore, healthcare professionals should be trained on the latest methods of identifying cases, controlling infections and protecting themselves from the disease.

**Conclusions:**

Despite being endemic to Madagascar, the outbreak’s pace is unparalleled, and it may spread to non-endemic areas. The utilization of a One Health strategy that encompasses various disciplines is crucial for minimizing catastrophe risk, antibiotic resistance, and outbreak readiness. Collaboration across sectors and proper planning ensures efficient and consistent communication, risk management, and credibility during disease outbreaks.

**Graphical Abstract:**

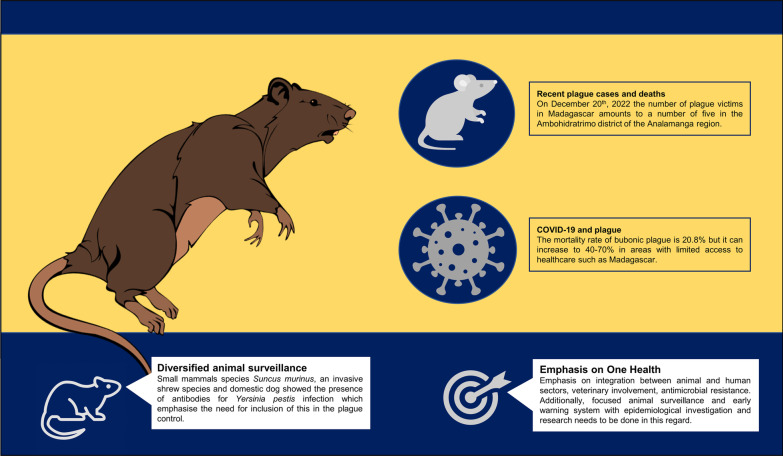

## Background

Since 1990s, the majority of human cases of plague have occurred in Africa. The countries with the highest incidence of plague are the Democratic Republic of Congo, Madagascar, and Peru [1]. Plague is a bacterial infection caused by *Yersinia pestis*. It has three forms, including bubonic, pneumonic, and septicaemic. The most common form is bubonic plague, which is characterized by the inflammation and swelling of the lymph nodes, also known as “buboes”. It is primarily transmitted from rodents to humans through flea bites, while human-to-human transmission of bubonic plague is uncommon but can occur if fleas are carriers [2].

Recent years have seen a rise in the frequency of plague outbreaks in Madagascar, attributed to various factors such as climatic changes, deforestation, population displacement, and resistance to treatment and control measures. These include rodent resistance to *Yersinia pestis*, flea resistance to insecticides, and the bacteria’s resistance to antibiotics [3]. The Central Highlands of Madagascar, with an altitude above 800 m, is a region where plague is commonly found and the transmission typically occurs between September and April. The Ministry of Public Health typically reports almost 200 and 400 instances of plague each year, the majority of which are in the bubonic form [4].

As of September 15th, 2021, the Ministry of Public Health has reported a total of 20 suspected and 22 confirmed cases of plague (19 with pneumonic plague and 3 with bubonic plague) [5]. These cases have been identified in two non-adjacent regions of the country: Arivonimamo district and Ambalavao district. Out of the confirmed cases 8 have resulted in deaths, 2 in bubonic plague cases and 6 in pneumonic plague cases resulting in a case fatality ratio of 37% [6]. According to the latest update on December 20th, 2022, three individuals have passed away due to illness, and three more are currently being hospitalized, including one man in critical condition, from the communes of Ambohimiadana, Antsaharasty, and Ampanotokana in the Ambohidratrimo district of the Analamanga region. The number of plague victims in this locality thus amounts to 5 [6].

## Transmission of plague in Madagascar

*Yersinia pestis*, the bacteria responsible for causing plague, can develop and be transmitted from three invasive species; the black rat (*Rattus rattus*), the brown rat (*Rattus norvegicus*), and the Asian house shrew (*Suncus murinus*) [7, 8]. The black rat is the primary reservoir of *Yersinia pestis* in rural areas where plague is most commonly found in Madagascar, while the brown rat is more commonly found in urban areas. Two species of fleas are known to play a role in transmitting *Yersinia pestis* in Madagascar: the oriental rat flea (*Xenopsylla cheopis*) which is found globally, and the endemic flea (*Synopsyllus fonquerniei*) which is primarily found in the Central Highlands [9].

## Plague deaths amid COVID-19 pandemic

From January 3rd 2020 to January 12th 2023, there were 67,760 confirmed cases of COVID-19, resulting in 1418 deaths [10]. Presently, the biggest concern is the potential plague spread among humans during the ongoing coronavirus disease 2019 (COVID-19) pandemic. The case fatality rate (CFR) of Bubonic plague stands at 20.8% with proper treatment, however in locations such as Madagascar, where superstitions, lack of financial resources, and limited access to medical facilities can impede the access to healthcare, the mortality rate can increase to 40–70% in the absence of treatment [11]. This is primarily due to the weakened state of the country’s healthcare system and limited access to healthcare in rural areas, which have been further exacerbated by the COVID-19 pandemic. A lack of access to healthcare facilities and inadequate levels of service provision, combined with the high cost of transportation and treatment, prevent 60% of people who report feeling unwell from seeking medical attention at a health centre [12]. Under-reporting of plague cases, which can lead to increased mortality rates, is primarily attributed to the combination of factors such as stigmatization, utilization of traditional healers and shortage of medicine at healthcare facilities often causes patients to forego seeking medical attention and instead rely on self-treatment, in addition lack of knowledge and awareness regarding the signs and symptoms of plague, particularly in rural areas is also one of the contributing factors [13].

## Potential strategies need to be adopted

### Strengthening of the Community Health Centre’s (CSBs)

In Madagascar, CSBs are the main point of access to healthcare services. However, due to a lack of funding, the healthcare system is under-resourced and only 60–70% of the population has access to basic primary healthcare [13]. Additionally, there are 86 district hospitals and 18 regional referral hospitals that provide more advanced diagnostic services, but the availability is limited. Healthcare services as well as trained manpower are more accessible in urban areas but are not evenly distributed, leading to limited access and affordability for those in disadvantaged neighbourhoods. There is a lack of trained healthcare staff and equipment resulting in inaccurate diagnoses and poor treatment outcomes, as well as shortage of medicine at healthcare centre’s [14]. Voluntary training of local leaders, healthcare providers as well as encouraging and sensitising them to actively participate and provide services in rural areas can significantly bring down the number of cases through early diagnosis and treatment.

### Robust vector, reservoir and pest control

Rodent populations and distribution may rise as a result of agricultural practises, deforestation, and wildfires, with a particular influence on migrants who are farming new acreage and are residing in substandard circumstances [9]. Low-income and marginalised people in Madagascar are disproportionately impacted by the illness and much more prone to come into encounter with rodents and fleas. The underlying socioeconomic causes of the plague’s spread must be taken into account for effective control methods. This entails tackling problems at the household and societal levels, such as poverty, subpar housing, and poor sanitation. Additionally, methods for reducing human-rodent interaction should be devised, such as secure food and granary storage and preventing animals from entering inhabited areas.

### Need for diversified animal surveillance and disease monitoring

The National Plague Control Program (NPCP) carries out regular monitoring for plague in human populations, examining all reported cases deemed suspicious. However, no corresponding program for monitoring plague in animals is in place. Studies have shown that small mammal species *Suncus murinus*, an invasive shrew species which does not develop plague disease, is a known reservoir of *Yersinia pestis* [7]. A 3-month-old domestic dog from the village of Ampanalana, which is located near a former plague quarantine hospital (Lazaret) in Toamasina, showed the presence of antibodies for *Yersinia pestis* infection. However, there is no evidence that the dog had developed symptoms of plague [15]. Emphasizes the possibility of utilizing animal surveillance to detect the potential transmission of plague in both endemic and non-endemic areas, for the purpose of targeted prevention and control measures.

### Need to address the research lacunae regarding the plague

There exist several scarcities in the literature that need to be addressed to gain a more comprehensive understanding of crucial contextual elements and to enhance the efficacy of community involvement and communication strategies pertaining to plague in Madagascar. It is imperative to investigate the healthcare seeking behaviour and the impact of funeral and burial customs on the transmission of plague. So that co-creative policies can be made keeping in mind the customary believes but also tracks the preparedness for outbreaks, which includes examination of protocols for body handling, initial interment, and practices that are deemed appropriate by the community.

### Need to implement One Health concept (OHC) for effectively managing the plague

Zoonotic diseases collectively have a significant impact on livestock production, food security, livelihoods, and environmental conservation. The broad-reaching effects and the interconnectedness of human, animal, and environmental health make it crucial for public health systems to employ a multi-disciplinary “One Health” approach that fosters coordination in order to better understand and manage the risks associated with these diseases.

#### Importance of OHC

(i) The reduction of catastrophe risk, the mitigation of antibiotic resistance, and the improvement of outbreak preparation planning may all be achieved through a “One Health” strategy. It can also enhance the assessment of sensitivity to the effects of climate change.
(ii) Early involvement of all stakeholders improves project success by promoting shared understanding, joint solutions, risk anticipation, gap addressing, redundancy reduction, and relevant coordination.
(iii) Consistent and effective communication, risk management, greater efficiency, and credibility maintenance during disease occurrences are ensured by good planning and strong collaboration across sectors.

## Approaches for implementation of One Health

**Fig. 1:**
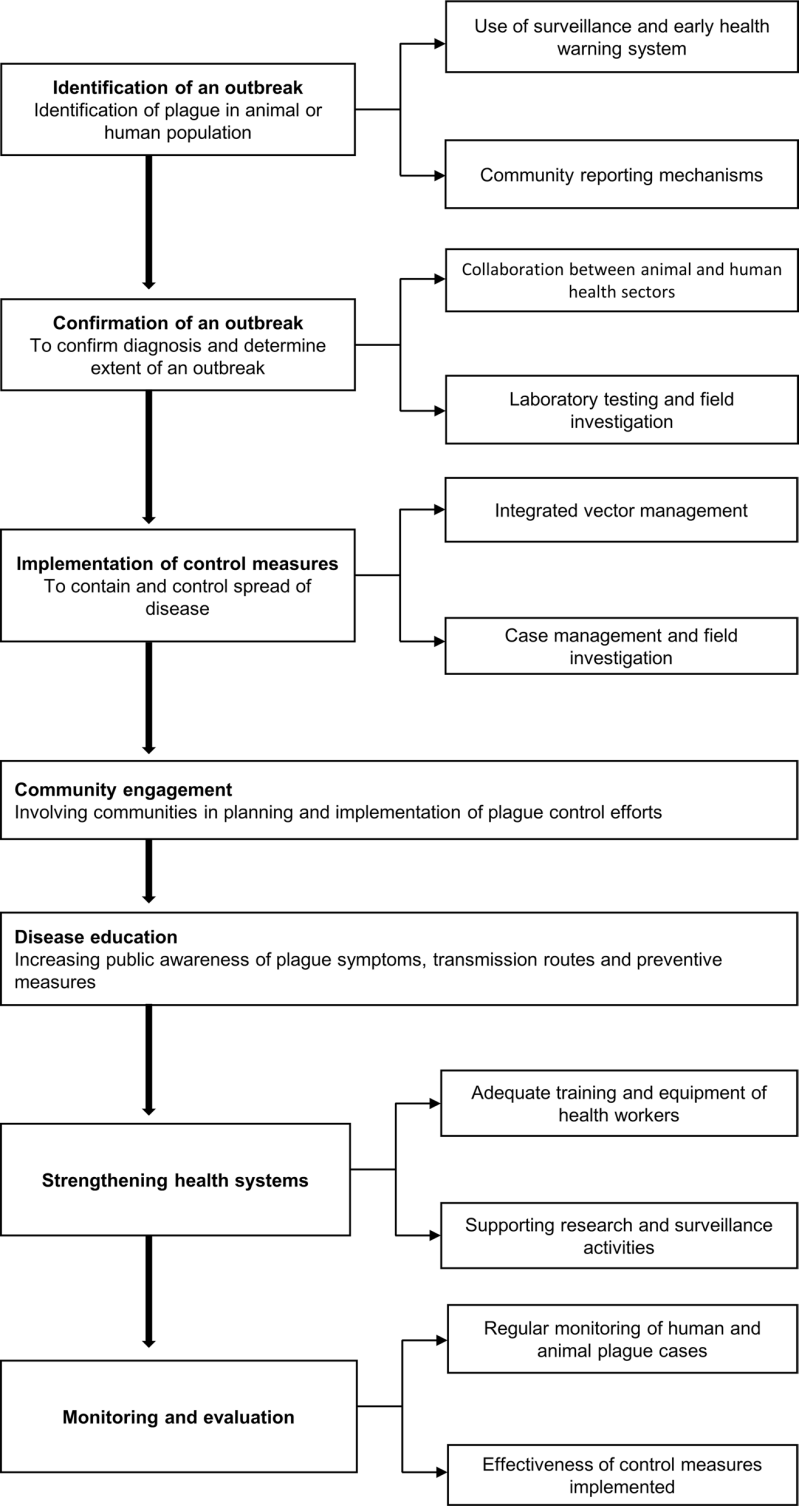
One Health model for prevention and control of plague

Adopting a One Health approach to controlling plague can be accomplished through a range of potential strategies which includes the following:

- Partnerships between human and animal health sectors: This strategy involves working together with veterinary professionals to identify and prevent outbreaks in animal populations, as well as educating the public on how to protect themselves from infection.
- Community involvement: Including communities in the planning and implementation of plague control efforts can increase their understanding of the disease and promote support for control measures. This can involve community based participatory approaches and engaging local leaders and traditional healers.
- Surveillance and early warning systems: Regular monitoring of plague cases in both human and animal populations can help quickly identify and respond to outbreaks [16]. This could involve the use of geographical information systems (GIS) mapping and real time data collection and analysis.
- Integrated vector control: This approach targets the fleas that transmit the disease to both humans and animals through a combination of chemical and non-chemical control methods.
- Disease education: Raising public awareness of plague symptoms, transmission routes, and prevention measures can help reduce the number of cases and deaths.
- Health system strengthening: This can be achieved by providing sufficient funding, training and equipment to health care workers, supporting research and surveillance activities, and ensuring availability of appropriate drugs and regimens (Fig. 1).

## Areas that need special emphasis in OHC

### Focussed animal surveillance for successful prevention and control

- Active surveillance of animal populations: This involves the systematic monitoring of animal populations, particularly those identified to be at a higher risk of plague transmission such as rats and other rodent species. This includes the collection and examination of samples for laboratory testing and identification of plague-positive animals.
- Community-based participatory approach: Engaging local communities in the identification and monitoring of animal populations, including training them to recognize signs of plague in animals and report cases to health authorities.
- Geospatial analysis: The utilization of GIS can be used to identify areas of high risk for plague transmission and to track the spread of the disease.
- Epidemiological investigations and evaluation: Ongoing research on the epidemiology of plague in the region, including identification of risk factors and examination of intervention strategies, can aid in enhancing the comprehension of the disease and guide the development and execution of future control measures.

### Special emphasis on antimicrobial resistance

- Antimicrobial management: Judicious utilization of antimicrobial agents in human and animal health, which includes the formation of recommendations for prescribing and dispensing antibiotics, as well as the monitoring of antibiotic resistance patterns.
- Antibiotic resistance surveillance: Ongoing monitoring of antibiotic resistance patterns in human and animal populations, as well as the gathering of information on the use and distribution of antimicrobial drugs.
- Investigations on genetic resistance: Studying and identifying genetic immunity in rodent populations to *Yersinia pestis* and comprehending the underlying mechanisms of immunity.
- Establishing a worldwide alliance to take concrete steps against antimicrobial resistance (AMR) is imperative. Meaningful advancements in the battle against AMR can only be achieved through global cooperation. It is crucial to raise awareness about AMR at the international level and tackle it through a One Health approach to bring about tangible changes [17].

## Conclusions

The lack of biological diagnostic laboratories in rural settings is a significant barrier to identifying the disease at an early stage. Isolating *Yersinia pestis* has been the traditional procedure for verifying the presence of the plague, which can take at least 4 days. But more recent fast diagnostic techniques have been created that can help with validation in areas where the plague is widespread. These tests must be provided more broadly available in rural regions as they provide new potential for tracking and controlling plague infections. Increased animal as well as human surveillance can aid in reducing the burden of the diseases which can be achieved through One Health. The number of instances would significantly reduce if the general population was made aware of the indicators, preventive, and infection control measures during funerals through various channels like as campaigns, posters, and social media. The most recent techniques for detecting instances, containing infections, and warding off sickness should also be taught trained to medical personnel. Despite being an endemic illness in Madagascar, this outbreak’s rapidity raises the chance that it will move to areas where the illness is less frequent.

## Data Availability

Not applicable.

## Abbreviations

COVID-19: Coronavirus disease 2019
CSBs: Centres de Santé de Base
CFR: Case fatality rate
NPCP: The National Plague Control Program
OHC: One Health concept
AMR: Antimicrobial resistance
